# How to Succeed in Our Profession

**Published:** 1880-04

**Authors:** 


### HOW TO SUCCEED IN OUR PROFESSION.

The first great element to success is a thorough education, to be sufficiently informed in leading sciences; but especially to be thoroughly posted on all branches of our profession, are marked requisites. One who accepts the responsibilities of a profession without such qualifications is unworthy the exalted relationship he seeks. The effort to lay first a broad basis for a professional superstructure may cost self-denial, self-sacrifice, toil and struggles; but the goodness of learning rightly rewards all who pay their devotions at her altars.

Professional prominence and business success are becoming every year more dependent upon a thorough collegiate education. If we consider our life-work in a mere business, financial sense, statistics show that educated mind has greatly the advantage, and that the time and money spent in colleges are both a saving of time and investment richly productive. Business men now bear testimony to the fact that education is of great service to merchants, publishers, mechanics, agriculturists, etc. In the profession there is every need for cultured mind. Only such rise to anything like commanding usefulness. Education is much more general now among the people, and they will demand more of all who strive to lead or instruct them. We are told that all professions and vocations are crowded; but there never was such a demand and such opportunities for thoroughly trained intellect and ripe scholarship. Hence many doors of usefulness and of pre-eminence open to every one giving any evidence of superior qualifications. Our profession is peer to any in offering facilities for acquiring a thorough knowledge of all her branches. A number of colleges are established here and there, presided over by the brightest lights, where all who aspire can go and be thoroughly cultured and skillfully trained. We have many journals, edited and contributed to by men of ability, and through them much valuable information can be gleaned.—*Dental Luminary.*

